# Family Caregivers Employed by Home Care Agencies: Lessons Learned From Switzerland and the United States

**DOI:** 10.3389/phrs.2023.1605849

**Published:** 2023-11-24

**Authors:** Iren Bischofberger, Mary Jo Vetter

**Affiliations:** ^1^ Institute of Nursing Science, Faculty of Social Sciences, University of Vienna, Vienna, Austria; ^2^ Rory Meyers College of Nursing, New York University, New York City, NY, United States

**Keywords:** family caregiving, home care, employment, Switzerland, United States

## Abstract

**Background:** Increasing demands for home care staff has been triggered in the past decades by shorter hospital length of stay, and a shift of responsibility for complex care regimens to private households. Therefore, an innovative model to employ family caregivers in home care agencies is expanding in Switzerland and the United States. This policy brief aims to identify core characteristics of the model and analyze its potential benefits and challenges.

**Evidence:** The model is expanding based on legal ground but without the requisite scientific evidence. After an initial patient assessment by a registered nurse, and assigned hands-on tasks to family caregivers, the salary is derived from payer reimbursement.

**Policy Options and Recommendations:** Standards need to be in place to determine the family caregivers qualification that are specific to the client situation of all age groups. Supervision of quality of care, labor law, and blurred roles of biographical relationships remains at the responsibility of the home care agency.

**Conclusion:** Further research for the data-driven exploration of the model is needed to inform the many stakeholders involved.

## Background

Internationally, family caregivers have long been the backbone of unpaid home care for individuals across the age continuum [1–3]. In Switzerland [4] and the United States [5] recent reports highlight this important role, provide guidance for future policy development, and make recommendations for policy action and informed decision making by healthcare providers and policymakers. The main focus of the recommendations is on “lightening the load” for family caregivers by calling for easier access to respite care, better reconciliation of employment and caregiving duties, and an increased recognition of family caregivers by healthcare professionals. The main objectives, in both countries, are to secure family caregivers’ health status before they experience negative health consequences [6]. The objectives become even more relevant due to shorter hospital length of stay, a shift of responsibility for complex care regimes to private households, and family caregivers’ growing participation in the paid labor force. In recent years, a workforce shortage of home based service providers, exacerbated by the COVID-19 pandemic, also has contributed to the problem. These trends are combined with changing gendered expectations regarding unpaid family caregiving, as well as an increasing number of families living further apart. Hence, the impact on family caregivers becomes more profound.

Therefore, an innovative staffing model has been operationalized in Switzerland [7], and in the United States [8]: employment of family caregivers by home care agencies. Hence, family caregivers become employees in the regular labor market (summary see Box 1). The aim of this policy brief is to present the staffing model’s core characteristics, to analyze potential benefits and challenges, to inform policymakers and healthcare providers, and to highlight the need for future research.

Box 1 **The model in summary**

- The tasks provided by an employed family caregiver in a single household are based on the patient’s initial needs assessment conducted by an RN from a state licensed home care agency.
- RN’s evaluate the family caregivers’ skills and preferences in relation to the patient’s physical, mental and cognitive healthcare needs and assign tasks to a family caregiver (mostly hands-on care, e.g., bathing, toileting, dressing). Other tasks (e.g., household chores) are not covered. Nursing-level care beyond CNA-level are provided by RNs.
- The salary is derived from payer reimbursement (in the United States: Medicaid; in Switzerland: shared by private health insurance and municipality).
- The employed family caregivers are protected by the home care agency’s liability insurance during the time they are on paid duty. Other tasks (e.g., household chores, rides to pharmacy, administration of finances) are provided during unpaid hours.
- The pay level depends on the home care agency standards (equal to non-family CAN or lower).
- In Switzerland, the number of paid hours per patient are usually limited to 60 h per 3 months, unless the home care service can prove that more hours are needed (e.g., in palliative care situations, for patients with severe neurological conditions).
- In the United States, each state has the power to create innovative healthcare delivery models by employing family caregivers under state Medicaid waivers to provide home and community based services, supporting patient and family autonomous decision making about the nature and location of service delivery.

## Evidence

To date, there is a considerable lack of research regarding the model. However, it is currently gaining attention in the home care industry in Switzerland and the United States [9, 10], partly in light of the trends named above, and partly because of increased policy awareness of family caregivers’ contribution to the healthcare system in both countries [4, 5]. The latter is mainly driven by staff shortages in healthcare. This gap between evidence and practice implementation provides momentum for this policy brief which will also consider the federally organized healthcare system regulations in both countries and the associated policy implications at the state level.

### Switzerland

In the year 2000, the model was first implemented in a few municipalities, predominantly in rural areas [11]. It is practiced in accordance with state regulations for licensed home care services. In the beginning, most home care agencies were reluctant to implement it due to the blurred role of family caregivers and some legal uncertainties regarding individuals who function as employees yet have a specific biographical closeness to the home care clients.

Some years later, in a landmark case in 2006, between a home care agency and a large private health insurance company, the Swiss Federal Supreme Court ruled that there is no difference between employing a spouse to provide care than employing any other home care staff without personal ties to the client [7]. The main reason for this decision was that home care agencies, as licensed service providers in a certain state, decide autonomously whom they employ. The court concluded that family members who are briefly instructed and deemed qualified by the agency can perform tasks (bathing, toileting, help for dressing, etc.) as long as they achieve the required quality of care. The personal relationship to the care-recipient is not decisive. Thus, if a family caregiver fulfills the home care agency’s requirements, they are to be treated as any other employee. Yet, the Supreme Court ruling also emphasized that there is no entitlement to be employed as a family caregiver. In fact, in some cases, declining employment might be indicated when the Registered Nurse (RN), who has conducted a needs assessment, is concerned about potential negative effects on the client [10]. The home care agency leadership has to guarantee the quality of care, as well as the RN’s continued supervision.

Regarding staff qualification, two differing positions are predominant: The Swiss Supreme Court requires family caregivers to be instructed by a RN for the specific tasks deemed necessary by the patient’s assessment. Yet, current practice requires employed family caregivers to complete a training to qualify for certification as a nurse assistant (CNA) (120 learning hours plus 12–15 days field experience). This requirement does not consider that the employed family caregiver works in a single household. CNAs are typically sent to different patients in various households. In order to reconcile the two differing positions, negotiations are underway to determine whether family caregivers must have a nursing assistant certification or another, more appropriate qualification specific to the family situation. This could be a flexible and individualized course based on the assessed competencies already acquired before the employment which are specific to the individual patient’s needs.

### United States

All 50 states and the District of Columbia offer self-directed Medicaid services for long term care [12]. These programs allow states to grant waivers to federal regulations that permit qualified individuals to manage their own long-term home-care services, as an alternative to the traditional model where services are managed by an agency. In some states, the beneficiary can elect to have an approved family member provide care [13, 14]. Paid family caregivers typically register with a home care agency who, in addition to payroll and benefits management, may provide clinical assessment, establishment of the plan of care, initial and ongoing caregiver education, and care management oversight. Generally, for a family member to be a paid personal care provider, a legally responsible relative has to be providing services that a parent or spouse would not be providing for a non-disabled spouse or minor child [15]. It is up to the state to define the specific circumstances under which relatives can be paid. Benefits, coverage, eligibility, and rules differ from state to state and can be complex and difficult for consumers to understand. States can choose to target specific populations or geographic areas for consumer-directed programs. The payment of family caregivers raises important questions for each state to consider such as oversight to ensure quality of care by persons who are trained, qualified, credentialed and meet background check requirements [8]. Successful models of family caregiving are addressing significant unmet home care service needs that lead to financial and emotional stress [9] and offer the opportunity to decrease unnecessarily long hospitalizations and institutionalization as family caregivers have better retention rates in spite of lower hourly wages [16]. Despite drawbacks related to caregiving burden, extra training, and equitable access to the model, families perceive it as highly valuable. The development of valid tools to measure the quality of home healthcare reported by families is underway to provide evidence to support further dissemination of the model [16].

## Policy Options and Recommendations

Based on the few literature sources, the employment model combines several benefits for family caregivers and home care agencies, yet it also has shortcomings.

### Benefits

Family caregivers are recognized as home care team members providing quality CNA-level care, and for this part of their care work, they are remunerated. They are protected by a work contract which regulates working hours, continuing education, and benefits such as vacation, sick time, and payments to pension and social security funds [11]. In addition, family caregivers have more flexibility in organizing the schedule of necessary tasks throughout the day. Being an employee of an agency allows family caregivers to be part of the team, and to be closer to decision making in care planning. Direct access to clinicians simplifies the communication processes [16]. For the home care agency, the employment model may serve as an additional option for family caregivers in addition to providing respite care. In case the employed family caregiver lives in the same household as the client or nearby, the home care agency can reduce time and cost for travel hours. Also, the model promotes enhanced continuity of care by reducing staff turnover compared to non-family CNAs [16]. At the societal level, in cases where family caregivers were not previously employed, the model generates income taxes and contributions to mandatory government funded programs such as social security.

### Shortcomings

Family caregivers’ household relationships are blurred when they combine unpaid work and tasks provided under the employment model leading to artificial separation of hours during the day or night into employed and non-employed caregiving times. Also, the required CNA course focuses on geriatric care, thus not meeting qualification needs of family caregivers who provide care to children or young adults [9]. The employment model covers a minor part of all family caregiving hours because the health insurance only allows a limited number of hours per month based on individual needs and existing healthcare policies. Also, depending on the economic sector, a change of job might lead to high opportunity costs for the employed family caregivers due to relatively low wages in the home care industry [10]. For the public, the model might lead to a shift of unpaid family caregiving provided altruistically towards a demand for more industry driven professional care with its associated regulations, rules and procedures. For the labor market, employed family caregivers might decrease their work activity in jobs in other economic sectors and increase staff shortages elsewhere.

The following policy recommendations links intertwined concepts: quality of care, educational requirements, and ethical considerations.

- The regulatory responsibility for quality of care remains with the home care agency for any client, whether cared for by an employed family caregiver or any other staff. Family caregivers might be particularly well suited as they are experienced in providing person-centered care, as many have provided unpaid care for years before they decide to be employed (e.g., progressive dementia). Others enter the employment as a caregiving novice, after a life altering health event of a loved one (e.g., stroke). In these two cases, experiences with caregiving tasks differ considerably. Hence, RN’s who conduct the needs assessment and meet with the client and their family caregiver in the home are in a pivotal position to evaluate the situation individually and target appropriate educational interventions according to identified needs. To date, no standardized family caregiver skills assessment systems are in place in either Switzerland or the United States. that take these variations into consideration. However, conceptual issues have been discussed in the past [17] recommending that a standard tool be used to determine specific educational requirements that are person-centric.
- In the Federal Supreme Court case from Switzerland, the caregiving spouse was represented as an unqualified care worker whose work effort did not have to be paid for by the health insurance basic package due to lack of basic qualifications. The Supreme Court, however, considered the home care agency as a service provider that needs to decide whom to employ. If a standardized CNA course was required, it does not consider the individual home care and client situation, and educational content is not provided at the location of care. Thus, CNA qualification might provide general information but not be relevant or effective in the employed family caregiver’s specific situation [9]. Thus, a modular educational approach provided in the private household is necessary to take into account the family caregiver’s established expertise.
- Biographical relationships between family caregivers and patients are sometimes disruptive due to violence or inappropriate behavior. These challenges are reported in consumer-driven models, where the client hires assistants who might be a family caregiver [18]. The employment model can serve as a protective system as it includes close supervision by RN’s and agency leadership who promote reflective practice to ensure workplace health for the patient and the employed family caregiver. Also, part-time employment is recommended, since one-to-one care for extended hours provided by a single-family caregiver might pose a risk of social isolation. The supervising RN must be alert to these issues and intervene according to professional standards.
- The blurred line between family caregiver and paid personnel can present challenges for interpreting labor laws given that family caregivers may provide services on and off the clock. This is especially important when the plan of care calls for more than a 40 h work week and the family relies on a single-family caregiver who may then be eligible for overtime pay. Though consumer directed programs in the United States have been demonstrated to be cost effective with built in controls through capitation or reimbursement methodologies [14], there is concern that states may struggle to maintain cost neutrality.
- Finally, home care teams can benefit from the genuine family caregiver perspective, which is fully integrated in the agency and triggers practitioners to be (more) family-centric [19].

## Conclusion

Based on the very limited available evidence, the employment model is not a “one-size-fits-all” solution for home care agencies and family caregivers. Yet, there is potential on micro, meso and macro system levels: On the micro level, family caregivers are supervised by an RN and protected by the healthcare agency and its human resource responsibility. Also, employment might increase family caregivers’ economic resilience [20]. On the meso level, home care agencies’ leadership is crucial to serve as early implementers for innovation such as new staffing models [16]. On the macro level, legal challenges may serve as an impetus for policy change. Also, family caregivers generate (more) income taxes and social insurance contributions, and family caregivers are better protected by social security benefits. Finally, policymakers, who are in charge of improving community based care, are triggered to think about innovation in home care provision.

In sum, the model certainly needs more conceptualization and research evidence, particularly on quality of care as well as cost and effectiveness before or while being rapidly upscaled. But the existing literature points to a promising start in a contemporary home care industry. At best, the existence of family caregivers in home care teams might foster a culture of family-friendly home care.
